# Early postoperative parastomal evisceration after explorative laparotomy: case report of a rare and potentially life-threatening surgical complication

**DOI:** 10.1186/s13037-023-00379-4

**Published:** 2023-10-23

**Authors:** Anis Hasnaoui, Racem Trigui, Sihem Heni, Salma Kacem

**Affiliations:** 1 Department of General Surgery, Menzel Bourguiba Hospital, Bizerta, Tunisia; 2 Faculty of Medicine of Tunis, Tunis El Manar University, Rue Djebal Lakhdar 100, Tunis, Tunisia

**Keywords:** Parastomal evisceration, Postoperative complication, Colon Surgery, Stoma, Surgical wound dehiscence

## Abstract

**Background:**

Parastomal evisceration represents a preventable surgical complication that should not occur with appropriate technical diligence and surgical skills. While late parastomal hernias are well described in the literature, there is a paucity of reports on the early postoperative occurrence of parastomal intestinal evisceration.

**Case presentation:**

An urgent laparotomy was performed on a 58-year-old female patient for an acute cecal perforation with generalized peritonitis related to underlying colon cancer. Intraoperative revelations necessitated a carcinologic right colectomy and the creation of an end-loop ileocolostomy. Following six sessions of adjuvant chemotherapy, Computed tomography scans raised uncertainties about the presence of peritoneal carcinomatosis. Consequently, a collaborative decision was reached in a multidisciplinary discussion to conduct a surgical biopsy of these deposits before reinstating digestive continuity. The surgical procedure started with stoma mobilization. However, adhesions and a relatively confined aperture curtailed a comprehensive peritoneal cavity exploration. Thus, a midline incision was executed. The verdict from the frozen section examination affirmed metastatic presence, prompting the retention of the stoma. Within 48 h post-surgery, an early-stage parastomal evisceration occurred, stemming from an inadequately sealed aponeurotic sheath. The exposed bowel surface was encased in fibrin, necessitating meticulous irrigation with a warm saline solution before repositioning it within the peritoneal cavity. Accurate adjustment of the aponeurosis closure ensued, coupled with a meticulous reconstitution of the stoma. The postoperative course was uneventful. The patient was subsequently referred for hyperthermic intraperitoneal chemotherapy.

**Conclusions:**

Preventing parastomal evisceration requires adherence to established stoma-creation protocols, including creating a properly sized fascial opening and secure fixation. In instances of excessive fascial opening, ensuring a tension-free and meticulous closure is imperative.

## Background

Parastomal evisceration represents a preventable surgical complication that should not occur with appropriate technical diligence and surgical skills [1]. The potential consequences of its negligence are dire, encompassing heightened morbidity and mortality rates attributed to the perils of bowel ischemia and necrosis [2]. While late parastomal hernias are well described in the literature, there is a paucity of reports on the early postoperative occurrence of parastomal intestinal evisceration [3]. This article serves to unveil a distinctive instance of early-stage parastomal evisceration. The peculiarity lies in its manifestation a mere 48 h into the postoperative course. The intent is to instill a heightened awareness among novice surgeons regarding the imperative nature of meticulously adhering to correct procedural protocols, irrespective of the surgical context.

## Case presentation

A 58-year-old female patient, known to have a medical history of type 2 diabetes and hypertension, was admitted to our surgical ward due to a perforated right colon tumor. She exhibited fever and tachycardia. Physical examination revealed a sizable mass, measuring 15 cm, and rebound tenderness in the right hemi abdomen. A digital rectal examination detected a painful bulging in the anterior wall of the rectum. In response to this critical condition, an emergency computed tomography (CT) scan was conducted. The results pointed towards a perforated cecal tumor along with two abscesses measuring 20 cm and 15 cm. These abscesses were located beneath the right diaphragmatic dome and within the Douglas pouch, respectively. There were no signs of metastatic spread observed. After a brief resuscitation, the patient underwent a median laparotomy. Initial assessment disclosed a generalized purulent peritonitis linked to the perforation of a 15 cm cecal tumor. Coexisting right subphrenic and Douglas abscesses were confined by the hepatic flexure and ileal loops, respectively. During the surgical procedure, the abscesses were appropriately drained, followed by a carcinologic right colectomy. Owing to the septic peritoneal environment, an end-loop ileocolostomy was created. Anatomopathological evaluation indicated a stage III colon cancer. The patient’s treatment plan extended to adjuvant chemotherapy. Subsequent evaluations through CT scans and abdominal magnetic resonance imaging revealed no evidence of liver metastasis but uncovered multiple cystic nodules dispersed throughout the peritoneal cavity. After multidisciplinary discussions, the decision was reached to proceed with a laparotomy, and frozen section examination of the cystic lesions prior to restoring digestive continuity. Notably, pre-surgery physical and radiological assessments did not reveal signs indicative of parastomal herniation.

**Fig. 1:**
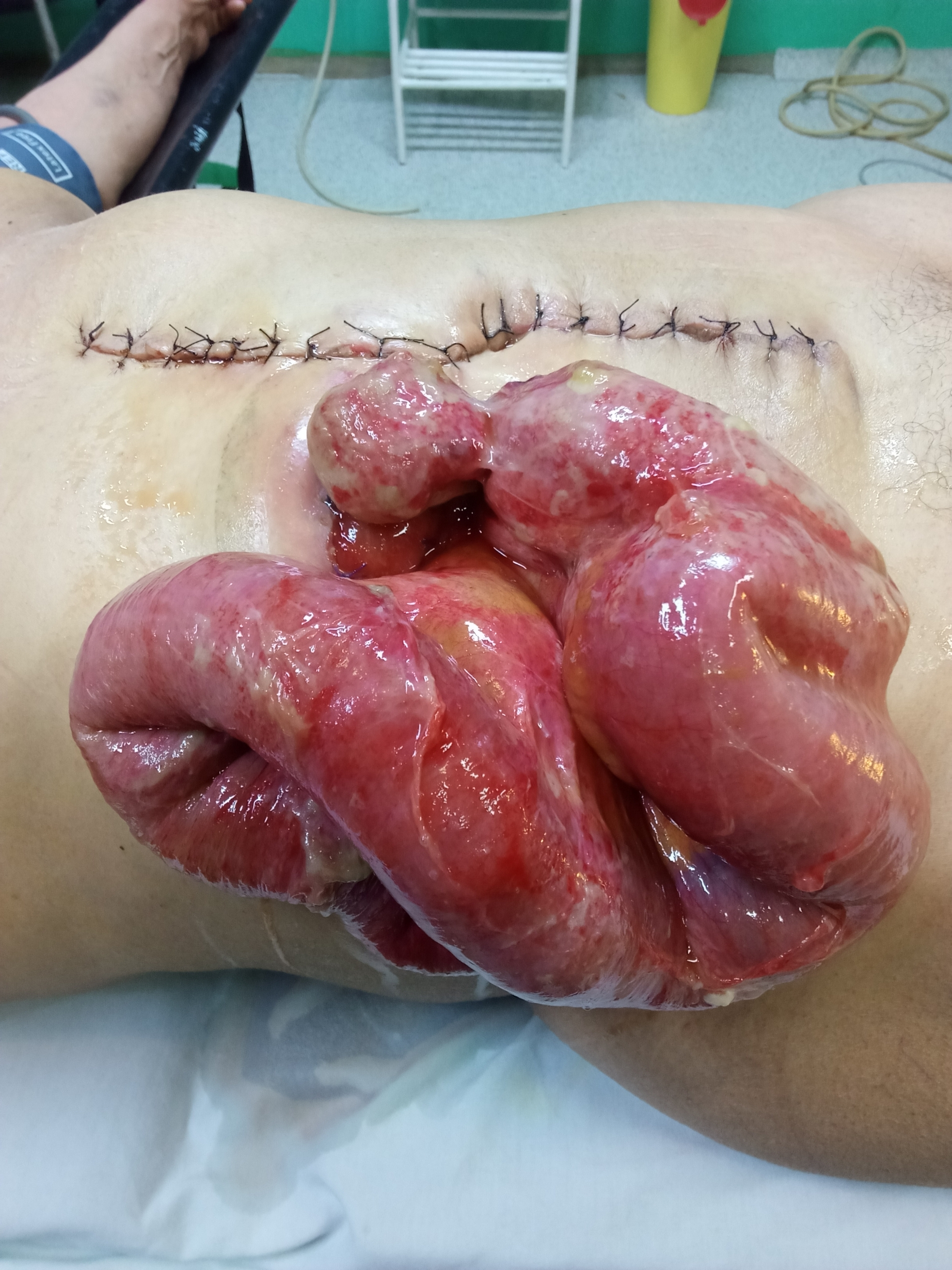
Parastomal evisceration. One meter of the small bowel eviscerated through the stoma orifice with mucocutaneous dehiscence

The patient underwent surgery one month after the last chemotherapy session. The surgical procedure started with stoma mobilization. However, adhesions and a relatively confined aperture curtailed a comprehensive peritoneal cavity exploration. Thus, a midline incision was executed. Intraoperative findings were indicative of carcinosis nodules dispersed throughout the peritoneal cavity, a conclusion corroborated by the frozen section report. To mitigate the risks of bowel obstruction and postoperative leakage, the decision was made to retain the double-barrel stoma. Within 48 h of the postoperative course, amid ongoing paralytic ileus, the patient reported abdominal pain and significant swelling at the stoma site. Upon assessment, it was revealed that approximately 1 m of the small bowel had eviscerated through the stoma location (Fig. 1). Immediate emergency surgery ensued, unveiling a large defect in the musculoaponeurotic sheath and a mucocutaneous dehiscence at the inferior angle of the stoma site. The exposed bowel surface was encased in fibrin, necessitating meticulous irrigation with a warm saline solution. Thankfully, the small bowel was viable, permitting the reduction of the herniated content through the expanded sheath defect. Ensuring the stoma’s secure placement, the sheath defect was meticulously addressed using slowly absorbable sutures. Similar suturing techniques were employed to close the skin incision, while mucocutaneous sutures were utilized to finalize the stoma setup. The immediate postoperative course was uneventful, and the patient was subsequently referred for hyperthermic intraperitoneal chemotherapy in a different establishment.

## Discussion

The term “stoma” refers to a segment of the digestive tract surgically connected to the anterior abdominal wall, allowing for the disposal of waste products, either on a permanent or temporary basis. Its etymology traces back to the Greek word “Stoma”, which translates to “mouth”. stomas have assumed a pivotal role in the surgical management of bowel neoplasms, with colorectal cancer reigning as the primary indication for intestinal ostomies [4]. This fact underscores their significance. Although surgeons perform intestinal stomas as a routine part of their responsibilities, this seemingly straightforward procedure harbors the potential for a diverse range of complications if not meticulously planned and executed. In a systematic review encompassing eighteen randomized controlled trials, which scrutinized stoma-related complications in adults, the cumulative incidence of such complications hovered around 26.5% [5]. Predominating among these were peristomal skin complications. Intriguingly, even though this study spanned over 1,000 patients across a span of 35 years, not a single instance of early parastomal evisceration was reported. Early parastomal evisceration constitutes a perilous postoperative occurrence that, if not addressed promptly, can trigger severe complications. The condition is so uncommon that its coverage in medical literature is confined to a handful of case reports. Considering its scarcity, several authors have pointed to the likelihood of underreporting of this particular postoperative complication [2]. A comprehensive survey of existing literature only yielded ten cases of early parastomal evisceration, as succinctly summarized in Table 1. Notably, the earliest occurrences were within the first 72 h of the postoperative period. To the best of our knowledge, the case we present stands as the inaugural instance of bowel evisceration through a stoma site within the initial 48 h after surgery.

**Table 1:** Reported cases of early parastomal evisceration

Case	Year of publication	First author	Demographic origin	Age	Gender	Stoma type	Indication	Date of the occurrence in the post-operative course	Patient-related Risk factors	Reported technical failure	Content of the evisceration
1	2021	Basnayake [4]	Siri Lanka	53	Male	Open surgery Loop ileostomy	Obstructive right colic cancer	Day 7	Asthma, malnutrition, disseminated malignancy in the peritoneal cavity	The disproportion between loop and stoma diameter	Ileal loop
2	2020	Mateæ [2]	Romania	84	Male	Open surgery Loop sigmoid colostomy	Obstructive rectal cancer	Day 3	Advanced rectal cancer, Important bowel distension	Late stoma maturation	Ileum
3	2019	Kulkarni [5]	India	50	Female	Open surgery Loop sigmoid colostomy	Recto vaginal fistula	Day 9	No identifiable risk factors	Not reported	Small intestine
4	2019	Kulkarni [5]	India	45	Male	Open surgery Loop sigmoid colostomy	Intra-operative rectal injury	Day 12	Chronic cough	Failure of aponeurotic close	Transverse colon and omentum
5	2017	Arbra [6]	USA	90	Male	Open surgery End ileostomy	Ogilvie syndrome	Day 7	Advanced age, mechanical ventilation	No technical failure was reported	Small intestine
6	2016	Salles [7]	Brazil	82	Male	Open surgery loop transverse colostomy	Obstructive Rectal cancer	Day 10	Advanced age, mechanical ventilation,	The disproportion between colostomy and aponeurotic diameter	Ileum
7	2016	Ramly [8]	USA	81	Male	Open surgery End ileostomy	Complicated pseudomembranous colitis	Day 9	Advanced age, malnutrition, systemic corticosteroids, COPD	Inadequate fascia and mucocutaneous fixation	Small intestine
8	2014	Azouz [9]	USA	69	Male	Open surgery End sigmoid colostomy	Necrotizing peri anal fasciitis	Day 3	COPD	Failure to close the aponeurotic wall	Small intestine
9	2011	Salles [10]	Brazil	62	Male	Open surgery loop transverse colostomy	Rectal cancer	Day 4	COPD, Mechanical ventilation	The disproportion between bowel and stoma diameter	Ileum and colon
10	2008	Fitzgerald [11]	UK	65	Male	Laparoscopic loop ileostomy	Rectal cancer	Day 10	No Identifiable risk factors	Inadequate fascia and mucocutaneous fixation	Ileum

COPD: Chronic obstructive pulmonary disease

**Fig. 2:**
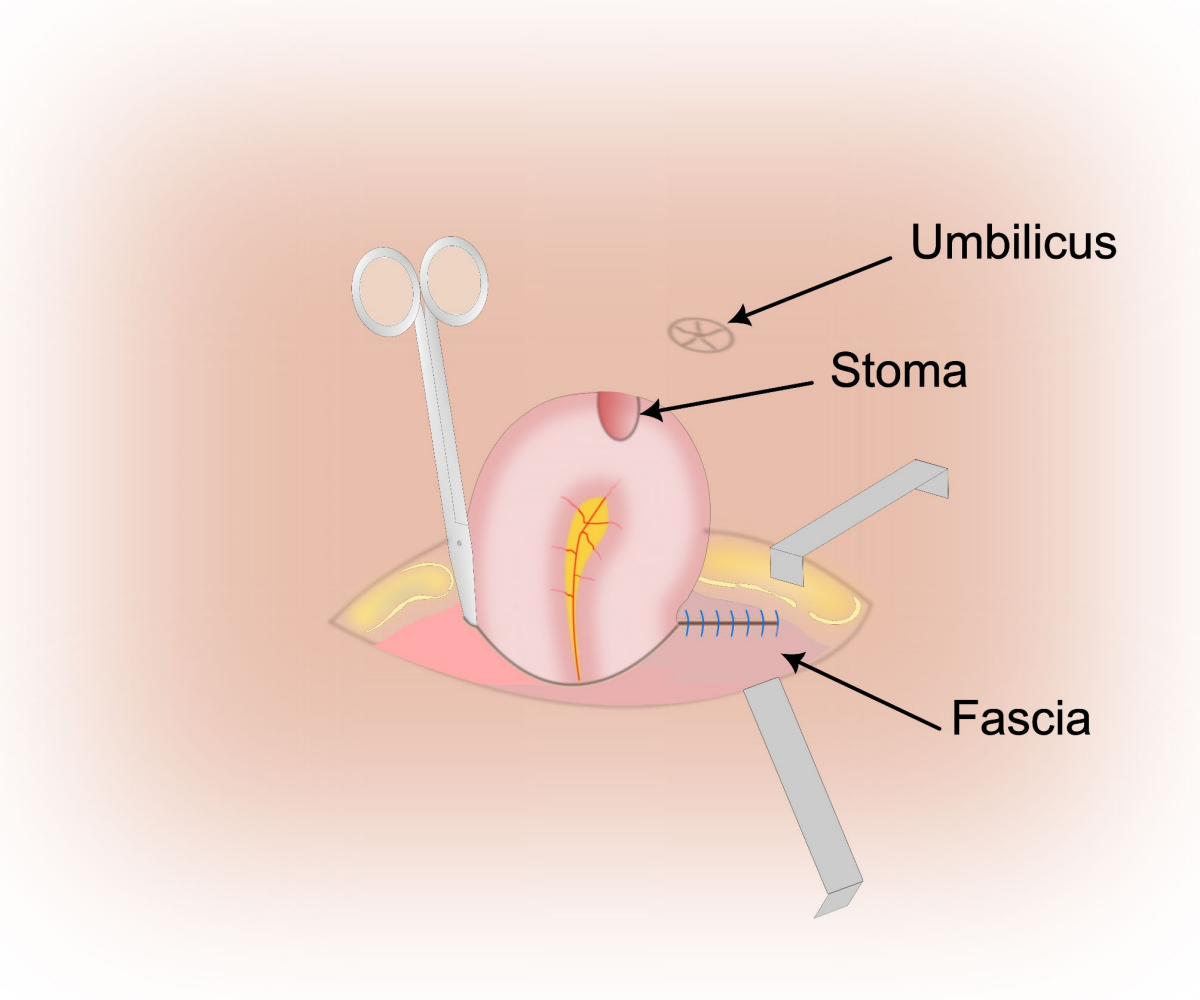
Closure of the aponeurotic sheath. The insertion of a closed Metzenbaum scissors through the aponeurotic opening in contact with the bowels ensures an adequate closure of the aponeurotic sheath (Neither tight nor loose)

The potential emergence of early parastomal evisceration in a patient’s postoperative course is contingent upon two distinct categories of risk factors: those intrinsic to the individual patient, arising from their medical and surgical history, and those associated with the execution of the surgical procedure itself. Patient-specific risk attributes, intricately tied to each patient’s medical background, can significantly influence the likelihood of parastomal evisceration. Elements within this category encompass advanced age, malnutrition, escalated intra-abdominal pressure elicited by factors such as chronic cough, prostatism, chronic obstructive pulmonary disease (COPD), or asthma. Moreover, the utilization of corticosteroids has also been identified as a pertinent risk factor [6]. The medical literature further implicates additional factors responsible for heightening intra-abdominal pressure, including disseminated malignancies, ascites, and instances of bowel obstruction within a previously explored peritoneal cavity [3]. Even the regression of intestinal edema could create enough space in the stoma site for the evisceration of intestinal loops [3]. Furthermore, it is widely acknowledged that surgical failures can often be attributed to technical challenges and the involvement of less experienced surgeons. These issues manifest as a disproportion between stoma diameter and the aponeurotic opening, ineffective aponeurotic stoma closure, inadequate fascia and cutaneous fixation, and delayed stoma maturation [2]. This underscores the critical role of meticulous surgical technique in mitigating the risk of complications. One of the pivotal steps in the creation of an ostomy is the fascial opening. It is advised to create a 3 to 4-centimeter cruciate incision in the anterior fascia. Following this, a vertical incision of equal length is made in the posterior rectus sheath after splitting the rectus abdominis muscle along its fibers [1]. In a recent experimental simulation study, Ambe explored the impact of a circular excision of the fascia in decreasing pressures at the ostomy site. This technique, proposed by the author, aims to diminish the risk of parastomal hernia [7]. In our comprehensive examination of published literature, the majority of cases (eight out of ten) displayed patient-related risk factors [2, 3, 6, 8–12], with two instances occurring without predisposing factors [9, 13]. Notably, pulmonary disorders like COPD and respiratory failure necessitating mechanical ventilation emerged as prominent patient-related risk factors. Meanwhile, our analysis of technical and surgical strategy failures yielded eight cases, with only one case showing no technical issues [9] and another lacking sufficient data [8]. Among these eight cases, the incongruity in diameter between the bowel and stoma site emerged as a recurrent concern [3, 10, 12]. In the context of our presented case, the patient’s advanced colon cancer, paralytic ileus, and prior peritoneal cavity exploration amplified the likelihood of this unfortunate post-operative event. However, akin to a substantial portion of cases in the literature, technical mishandling, specifically aponeurotic sheath closure failure, stood out as the focal point of this complication. The approach to addressing evisceration hinges on the viability of the affected bowel. Prompt intervention is imperative to forestall intestinal ischemia and infarction. The surgical course involves expanding the hernia opening, carefully reducing the eviscerated bowel, and possibly necessitating resection of necrotic tissue. Stoma revision and proper closure of the aponeurotic sheath (Fig. 2) are fundamental steps in this. A tension-free closure is key to reduce the risk of surgical wound dehiscence. When needed, relaxation incisions on the anterior aponeurotic sheath, on both sides of the suture line, could help reduce tension. In cases of concomitant peritoneal carcinomatosis, the use of a mesh could potentially add complexity to a planned cytoreductive surgery [14].

## Conclusions

Parastomal evisceration is a potentially life-threatening complication. Preventing this condition requires strict adherence to established stoma-creation protocols. An adequate fascial opening tailored to the stoma’s size, along with secure fixation, are pivotal stages in this process. In instances of excessive fascial opening, ensuring a tension-free and meticulous closure is imperative. Although prophylactic mesh placement may be considered to avert this complication, it is not advised in cases of peritoneal carcinomatosis with scheduled cytoreductive surgery. Instead, relaxation incisions should be implemented as a more suitable alternative.

## Data Availability

Not applicable.

## Abbreviations

CT: Computed tomography
COPD: Chronic obstructive pulmonary disease

## References

[1] Whitehead A, Cataldo PA (2017). Technical considerations in Stoma Creation. Clin Colon Rectal Surg.

[2] Mateş IN, Gheorghe M, Tomşa R, Sumedrea EL (2020). Paracolostomy evisceration: short review and a New Case Report. Chir Buchar Rom 1990.

[3] Basnayake O, Prasanthan Y, Jayarajah U, Ganga N, De Silva K (2021). Early parastomal evisceration of small bowel following a loop ileostomy for malignant intestinal obstruction. SAGE Open Med Case Rep.

[4] Ambe PC, Kurz NR, Nitschke C, Odeh SF, Möslein G, Zirngibl H (2018). Intestinal ostomy. Dtsch Arzteblatt Int.

[5] Malik T, Lee M, Harikrishnan A (2018). The incidence of stoma related morbidity – a systematic review of randomised controlled trials. Ann R Coll Surg Engl.

[6] Ramly EP, Crosslin T, Orkin B, Popowich D (2016). Strangulated ileostomy evisceration following lateralizing mesh repair of parastomal hernia. Hernia.

[7] Ambe PC (2021). The safety of surgical technique for ileostomy and colostomy in preventing parastomal hernias: an in vitro experimental simulation study. Patient Saf Surg.

[8] Kulkarni AA, Chauhan V, Sharma V, Singh H (2019). Parastomal evisceration: a report of two cases and review of literature. Cureus.

[9] Arbra CA, Fann SA (2017). Parastomal evisceration: rare complication after total abdominal colectomy. Am Surg.

[10] Salles VJA, Mardegan C, Pereira MJM, Giovanni P. Intestinal Extrusion by the Hole of Colostomy. 2016;3.

[11] Azouz V, Simmons JD, Abourjaily GS (2014). Immediate postoperative parastomal end sigmoid hernia resulting in evisceration and Strangulation. J Surg Case Rep.

[12] Salles VJA, Saba E, Pissinin ER, Arguello ERF, Machado Filho HN (2011). Complication related to colostomy orifice: intestinal evisceration. J Coloproctology Rio Jan.

[13] Fitzgerald JEF, Tang SW, Lake EJ, Richards T, Acheson AG (2008). Small bowel evisceration: a rare complication of laparoscopic ileostomy. Colorectal Dis off J Assoc Coloproctology G B Irel.

[14] Sugarbaker PH. Hernia Mesh is Contraindicated in Patients with Peritoneal Metastases. JSM Surg Proced [Internet]. 2018 [cited 2023 Oct 8]; Available from: https://www.jscimedcentral.com/article/Hernia-Mesh-is-Contraindicated-in-Patients-with-Peritoneal-Metastases.

